# Demand for CT scans increases during transition from paediatric to adult care: an observational study from 2009 to 2015

**DOI:** 10.1259/bjr.20170467

**Published:** 2017-12-08

**Authors:** Pete Thurley, Jonathan Crookdake, Mark Norwood, Nigel Sturrock, Andrew W Fogarty

**Affiliations:** 1 Royal Derby Hospital, Derby, UK; 2 Division of Epidemiology and Public Health, University of Nottingham, Clinical Sciences Building, City Hospital, University of Nottingham, Clinical Sciences Building, City Hospital, Nottingham, UK

## Abstract

**Objective:**

Avoiding unnecessary radiation exposure is a clinical priority in children and young adults. We aimed to explore demand for CT scans in a busy general hospital with particular interest in the period of transition from paediatric to adult medical care.

**Methods:**

We used an observational epidemiological study based in a teaching hospital. Data were obtained on numbers and rates of CT scans from 2009 to 2015. The main outcome was age-stratified rates of receiving a CT scan.

**Results:**

There were a total of 262,221 CT scans. There was a large step change in the rate of CT scans over the period of transition from paediatric to adult medical care. Individuals aged 10–15 years experienced 6.7 CT scans per 1000 clinical episodes, while those aged 19–24 years experienced 19.8 CT scans per 1000 clinical episodes (*p* < 0.001). This difference remained significant for all sensitivity analyses.

**Conclusion:**

There is almost a threefold increase in rates of CT scans in the two populations before and after the period of transition from paediatric to adult medical care. While we were unable to adjust for case mix or quantify radiation exposure, paediatricians’ diagnostic strategies to minimize radiation exposure may have clinical relevance for adult physicians, and hence enable reductions in ionizing radiation to patients.

**Advances in knowledge:**

A large increase in rates of CT scans occurs during adolescence, and considering paediatricians’ strategies to minimize radiation exposure may enable reductions to all patients.

## INTRODUCTION

The use of ionizing radiation in medical imaging is an important medical advance that has positively contributed to the diagnosis and management of many diseases. As a consequence of increased availability, CT scanning rates are increasing, as demonstrated by the recent report that the use of CT scans to evaluate respiratory symptoms has quadrupled over the past decade in the USA.^1^ However, exposure to ionizing radiation is well recognized to result in an increased risk of subsequent cancer;^2^ a risk that is higher in younger individuals owing to enhanced susceptibility and longer remaining life expectancy compared to an older population.^3–5^ The Image Gently Alliance is a worldwide coalition of healthcare organizations, founded by American societies, but with alliance organizations around the world, including the British Society of Paediatric Radiology.^6,7^ They conclude that the long-term risk of CT is small but real and important enough to change practice. The optimal imaging strategies recommended for children are also relevant in young adults. New imaging algorithms are permitting reductions in radiation dose while maintaining CT scan image integrity,^8,9^ and paediatricians in particular are encouraged to develop and use diagnostic strategies that avoid ionizing radiation if possible.^10–12^

At the age of 16 to 18 years,^13^ clinical care in the UK is transferred to adult physicians. We used 7 years of data from a busy general hospital in the UK to explore trends in demand for CT scanning stratified by age, and tested the hypothesis that rates of CT scans increased over the period of transition from paediatric to adult care.

## METHODS AND MATERIALS

### Study population

The Royal Derby Hospital (anonymized) is a busy teaching hospital in the Midlands of the UK, which provides clinical care for the local population. It received a total of 1,016,340 inpatient, outpatient and emergency department visits in 2015. Data were obtained on all CT scans performed from 2009 to 2015 stratified by age, along with comparable data on total clinical activity. As this was an analysis of routinely collected anonymized summary data, no ethical approval was required after review by the Department of Research and Development.

### Data analysis

The data were used to generate annual rates for the number of CT scans for each age group (numerator) as a fraction of the total number of patient episodes (denominator). The primary outcome was the difference between the rates of individuals who received a CT scan seen in 5-year age ranges that spanned the period of transition from paediatric to adult care from 16 to 18 years using an unpaired *t*-test. Sensitivity analysis compared the rates of CT scanning for consecutive 5-year periods using cut-offs of 16, 17 and 18 years.

## RESULTS

**Figure 1.**
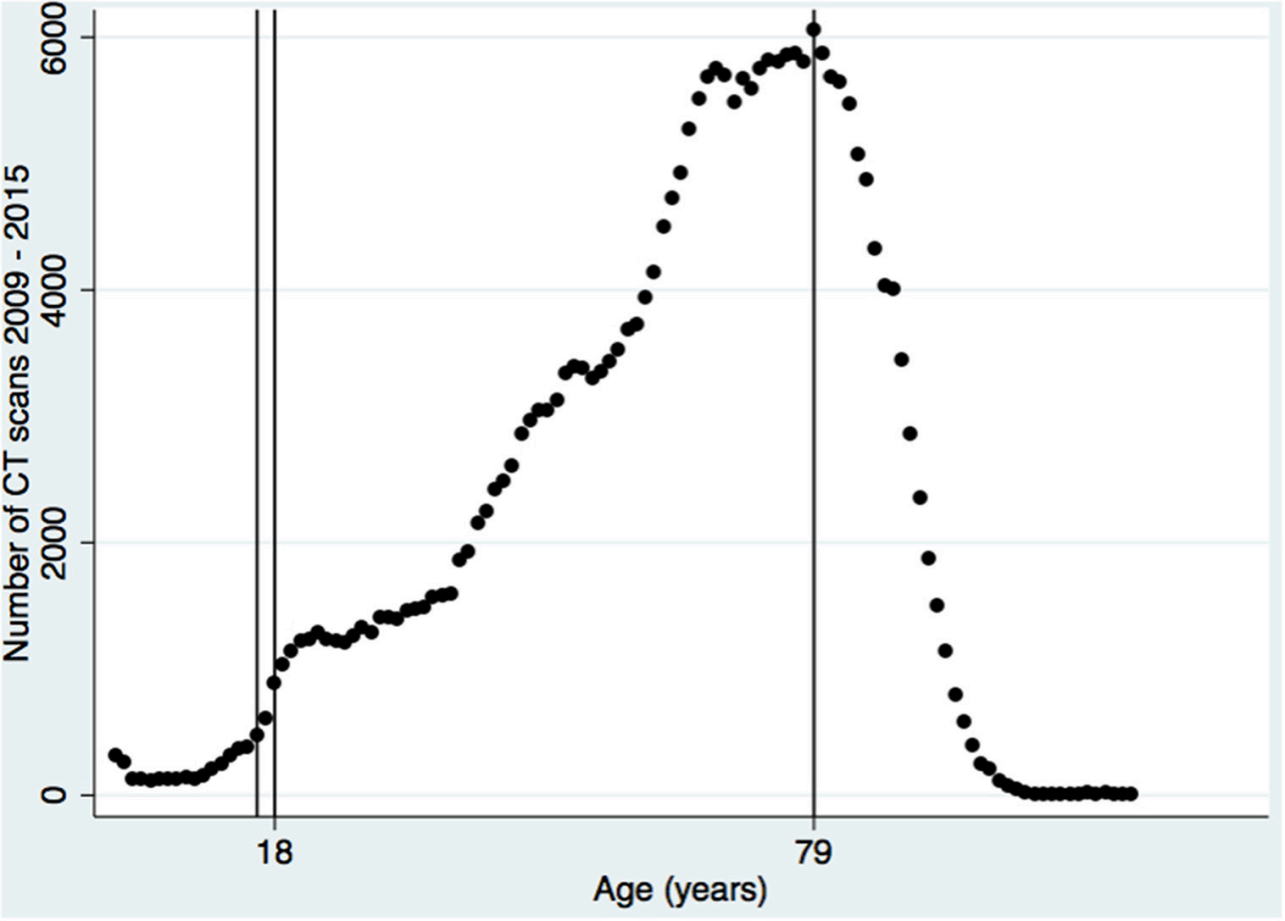
Absolute numbers of CT scans performed at the Royal Derby Hospital from 2009 to 2015 stratified by age. Vertical lines represent ages of 16, 18 and 79 years old respectively.

Over the study period there were a total of 262,221 CT scans, with a maximum number performed at the age of 79 years (Figure 1). In 2015, there were a total of 52 CT scans performed on 15 year olds and 149 scans on 19 year olds.

**Figure 2.**
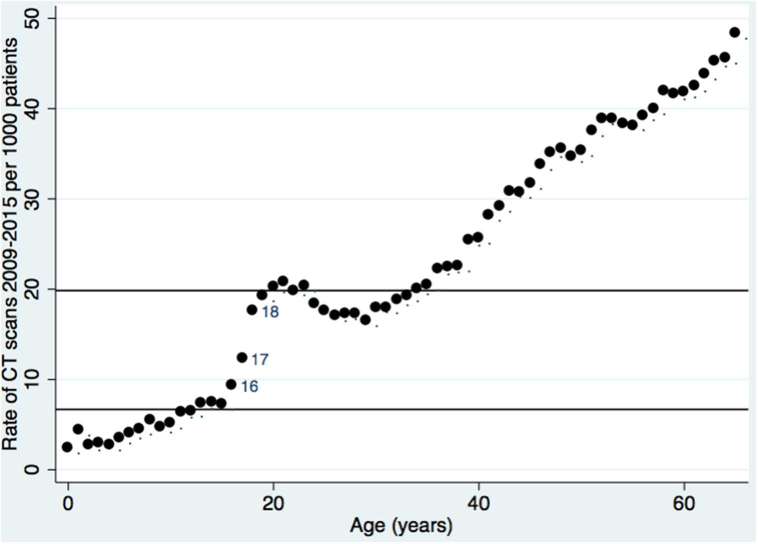
Rates of CT scans performed at the Royal Derby Hospital from 2009 to 2015 in adults aged less than 65 years stratified by age. Horizontal lines represent the mean CT scan rates for age Group 10–15 years (6.7/1000 patient episodes) and 19–24 years (19.8/1000 episodes).

The age-stratified rates of CT scans are presented in Figure 2. This demonstrates a clear step-change over the period of transition from paediatric to adolescent services at the ages of 16 to 18 years. The mean rate for 10- to 15-year-old children was 6.7/1000 patient episodes while the mean rate for 19- to 24-year-old adults was 19.8/1000 patient episodes (*p* < 0.001, unpaired *t*-test). This difference of 13.1/1000 was diminished but remained highly statistically significant for comparisons of the rates of CT scanning for consecutive 5-year periods using cut-offs of 16, 17 and 18 years (data not presented).

## DISCUSSION

We have demonstrated an almost a threefold increase in rates of CT scanning for individuals at the end of the period of transition from paediatric to adult medical services, compared to before this period begins.

The strengths of these data are that they represent complete data from a general hospital that is likely to be broadly representative of UK hospitals as a whole, and hence likely to be generalizable to other hospitals in the UK. While the models of delivery of healthcare vary in the UK compared to other developed countries, concerns about unnecessary and avoidable exposure of children and young people to radiation are global. We anticipate that our observations will be relevant and important to all healthcare providers that utilize CT scans. Although the increase in the rate of CT scanning over the period of transition from paediatric to adult medical services is clearly evident visually in the graphs, the consistency of this observation across extensive sensitivity analyses gives confidence that this is not a consequence of selective definitions of age groups but a real difference.

Our data are limited by the lack of detail on type of CT scan, the indication for the CT scan and the radiation dose delivered during the imaging process. This was because the study population at risk of having a CT scan (or denominator) was everyone who attended the hospital, as this allowed confidence in the data integrity over the 7 years for which measures of clinical activity were available. By generating rates of CT scans (number of CT scans/total number of patients seen) stratified by the age of population at risk, we were able to construct summary statistics that allow for changes in clinical activity over the period of the study, and also permit other investigators to compare their data with our standardized outcome measure. However, this approach becomes limited when considering further subcategorization of these data. This is because there are many permutations of CT scan (*e.g.* chest only v Chest, Abdomen and Pelvis) that prevent meaningful categorization, the indication for the CT scan is given in free text and is not available for epidemiological analysis, and the radiation dose is likely to have changed over the period of the study as CT scan methodology has improved. Subgroup analysis by referring speciality would also be challenging to deliver with certainty as many patients who receive CT scans will be under more than one clinical team, and hence generating a reliable denominator for the population at risk is impossible. An alternative approach would be to study many other similar hospitals to investigate if these age-stratified differences in exposure to CT scan ionizing radiation are the same elsewhere, or if there is heterogeneity between different centres.

Future studies may wish to study these variables, as this may help identify which patient groups are driving these age-stratified differences in CT scan rates, and may hence help understand and possibly modify clinical practice. The Royal Derby Hospital does not contain a major trauma centre, but adolescents in the UK can drive a moped (small motorbike) at 16 years and a car at 17 years. Hence, we cannot exclude the possibility that some of our age-related differences in CT scan rates are a consequence of trauma from use of motorized transport.

The large increase in rates of CT scanning over the period of transition from paediatric to adult services is unlikely to be a consequence of a comparable increase in rates of disease, but may reflect difference in clinical practice between paediatricians compared to adult physicians. In particular, paediatric training emphasizes reduction in unnecessary radiation exposure and as a consequence promotes non-ionizing diagnostic strategies where possible.^10,11^ Our experience as adult physicians is that while education on the risks of radiation and how to minimize exposure is acknowledged for our patients, it may be a lower priority than in paediatrics. This study was prompted by the wry comment that doctors act as if the risks of ionizing radiation drop on a patient’s 18th birthday, and these data are broadly consistent with this hypothesis.

It is likely that adult physicians can learn from their paediatric colleagues with regard to making awareness of the risks of exposure to ionizing radiation a higher priority in both initial training and also continuing medical education. Other strategies that can also be used include “nudge” theory that opportunistically uses passive interventions to inform professionals and aims to improve patient care. One option would be to use the CT scan report to inform the requesting professional of the probability of risk of cancer that results from CT scan exposure, which may improve radiation awareness of the risks of radiation exposure for subsequent clinical practice. A modified version of this approach of the cost-feedback on diagnostic tests reduced demand over 1 year by 32%,^14^ and subsequent cancer risk as a consequence of ionizing radiation exposure can be considered one of the costs of a CT scan. An alternative approach would be to inform the requesting physician of the cancer risks at the time of ordering the scan using electronic health care systems. However, we consider that this is unlikely to be as effective in the UK as the tests are generally requested after the decision to order the CT scan has been made with the patient, and the in-patient tests are often ordered by a junior doctor rather than the senior physician who made the decision to request the CT scan for the patient. At the institutional level, the ratio of number of CT scans at ages 15 and 18 provide a measure that may permit comparison of similar medical centres, as well as evaluate the effectiveness of radiation reduction interventions over time.

While our data demonstrate a substantial increase in the number of CT studies performed in patients around the time of transition from paediatric to adult services, it is difficult to quantify the clinical significance of this. Firstly there is uncertainty over the absolute risk of radiation exposure, although there are data from both atomic bomb survivors and from direct epidemiological studies of paediatric CT which are consistent in suggesting there is a very small (but unlikely to be zero) risk from the dose received when undergoing a CT scan.^15^ In addition to this, advances in CT technology have led to a decrease in radiation exposure, with the dose from paediatric CT studies reported to have halved over the period between 1990 and 2000^16^. During the period of our study further advances including tube current modulation and automatic exposure control have allowed an additional decrease in dose without sacrificing diagnostic information.^17^ Further refinements such as the use of more advanced image reconstruction techniques, owing to the ability to process algorithms with higher mathematical complexity in a timely manner, will continue to decrease the radiation burden to patients.^18^ Despite this, given the uncertainty over the long-term effects of radiation in the young population we have identified in this study, clinicians should still be mindful of the potential implications of radiation exposure.

In conclusion, these data from a busy UK teaching hospital demonstrate that the rate of CT scans in are almost three times higher in individuals who are old enough to have completely transitioned to adult medical care compared to those who are too young to have begun this process. A contributing factor is likely to be differences in clinical practice between adult physicians compared to paediatricians, and improving awareness of the risks of ionizing radiation in the former group has potential to reduce the exposure of a susceptible group of individuals to radiation in the UK. This in term will decrease their risk of cancer in later life.^4^ However, as CT scans are a fundamental tool for clinical diagnosis, it should also be emphasized that they are often the best imaging choice to ensure delivery of optimal medical care.

## FUNDING

Health Foundation’s Behavioural Insights Programme.
